# Influence of force field choice on the conformational landscape of rat and human islet amyloid polypeptide

**DOI:** 10.1002/prot.26432

**Published:** 2022-10-07

**Authors:** Sandra J. Moore, Evelyne Deplazes, Ricardo L. Mancera

**Affiliations:** ^1^ Curtin Medical School, Curtin Health Innovation Research Institute, Curtin Institute for Computation Curtin University Perth Western Australia Australia; ^2^ School of Chemistry and Molecular Biosciences The University of Queensland St Lucia Queensland Australia

**Keywords:** intrinsically disordered proteins, islet amyloid polypeptide, metadynamics, secondary structure

## Abstract

Human islet amyloid polypeptide (hIAPP) is a naturally occurring, intrinsically disordered protein (IDP) whose abnormal aggregation into toxic soluble oligomers and insoluble amyloid fibrils is a pathological feature in type‐2 diabetes. Rat IAPP (rIAPP) differs from hIAPP by only six amino acids yet has a reduced tendency to aggregate or form fibrils. The structures of the monomeric forms of IAPP are difficult to characterize due to their intrinsically disordered nature. Molecular dynamics simulations can provide a detailed characterization of the monomeric forms of rIAPP and hIAPP in near‐physiological conditions. In this work, the conformational landscapes of rIAPP and hIAPP as a function of secondary structure content were predicted using well‐tempered bias exchange metadynamics simulations. Several combinations of commonly used biomolecular force fields and water models were tested. The predicted conformational preferences of both rIAPP and hIAPP are typical of IDPs, exhibiting dominant random coil structures but showing a low propensity for transient α‐helical conformations. Predicted nuclear magnetic resonance Cα chemical shifts reveal different preferences with each force field towards certain conformations, with AMBERff99SBnmr2/TIP4Pd showing the best agreement with the experiment. Comparisons of secondary structure content demonstrate residue‐specific differences between hIAPP and rIAPP that may reflect their different aggregation propensities.

**Abbreviations**
2D‐IR: two‐dimensional infrared
BEMD: Bias exchange metadynamics
CD: circular dichroism
cMD: conventional molecular dynamics
CV: collective variable
DSSP: Define Secondary Structure of Proteins
hIAPP: human islet amyloid polypeptide
IAPP: Islet amyloid polypeptide
IDP: intrinsically discorded protein
IM‐MS: Ion mobility mass spectrometry
MD: molecular dynamics
NMR: nuclear magnetic resonance
REMD: replica exchange molecular dynamics
REST2: replica exchange with solute tempering
rIAPP: rat islet amyloid polypeptide
RMSD: root mean square deviation
T2D: type‐2 diabetes

## 1 INTRODUCTION

Islet amyloid polypeptide (IAPP) or amylin is a key hormone implicated in the development of type 2 diabetes (T2D). IAPP is stored in the pancreatic β‐cells along with insulin secretory granules and plays a role in the endocrine system and glucose regulation by slowing gastric emptying, reducing gastric secretion, and promoting satiety.^1^ The peptide is a 37‐residue long, intrinsically disordered protein (IDP), which means that it is highly conformationally flexible and lacks a well‐defined secondary and tertiary structure.^2^ In addition, IAPP is amyloidogenic, and its soluble monomers can aggregate into soluble, disordered oligomers, which then form insoluble, ordered amyloid fibrils (stacked β‐sheets).^3^ Both the oligomeric and fibrillar forms of IAPP are toxic to pancreatic β‐cells.^4^, ^5^ As IAPP is both an IDP and amyloidogenic, it is difficult to characterize its solution structure using structure determination methods such as nuclear magnetic resonance (NMR) spectroscopy, circular dichroism (CD), or two‐dimensional infrared (2D‐IR) spectroscopy. As a result, the structure of IAPP in solution in physiological conditions is unknown.

Most studies on IAPP have focused on comparing human IAPP (hIAPP) and rat IAPP (rIAPP). The two peptides differ by six residues (Table 1); however, rIAPP aggregates to a much lesser extent and T2D is not observed in rats. Three of these amino acid differences in rIAPP involve replacement with Pro, referred to as a “secondary structure breaker.”^.^ ^6^, ^7^ A central region in hIAPP (residues Ser20–Ser29) is thought to be responsible for beginning aggregation, with five of the six residues that are different between hIAPP and rIAPP occurring within this region.^8^ Transgenic mice studies have confirmed that these amino acid differences are responsible for the distinct tendencies for aggregation: homozygous transgenic mice with high expression of hIAPP developed amyloid deposits, resulting in the spontaneous development of T2D.^9^ Consequently, understanding the structural differences between rIAPP and hIAPP could shed light on the structure–activity relationship of IAPP and its aggregation properties.^10^, ^11^

**TABLE 1:** *(*A) Sequence of hIAPP and NMR structure ensemble of hIAPP in a micelle environment (PDB code 2 L86)^19^; (B) Sequence of rIAPP and the NMR structure ensemble of rIAPP in a micelle. Environment (PDB code 2KJ7)^18^

A: hIAPP K(CNTATC)ATQRLANFLVHSSNNFGAILSSTNVGSNTY‐NH2	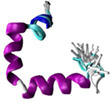
B: rIAPP K(CNTATC)ATQRLANFLVRSSNNLGPVLPPTNVGSNTY‐NH2	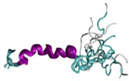

*Note*: Differences in the sequence of rIAPP with respect to hIAPP are highlighted in red. The Cys (C) residues forming a disulfide bridge are annotated by brackets.

Abbreviations: hIAPP, human islet amyloid polypeptide; NMR, nuclear magnetic resonance; rIAPP, rat islet amyloid polypeptide.

NMR secondary chemical shifts and CD spectroscopy data suggest that monomeric rIAPP in solution exhibits predominantly random coil conformations even after prolonged periods, reflecting its lack of tendency for aggregation into insoluble fibrils.^12^, ^13^, ^14^ Nonetheless, the values of the Hα, Cα, and C=O secondary chemical shifts also reveal that residues Ala5‐Ser19 transiently sample α‐helical conformations and that this α‐helical propensity is not sensitive to changes in temperature.^14^ The disulfide bond between Cys2 and Cys7 likely limits the tendency for a well‐defined secondary structure in the N‐terminal region. Similar structural features have also been inferred from IR spectroscopy data reported by Reddy et al.,^15^ whose analysis of amide hydrogen absorption bands suggested the presence of both α‐helical and random coil conformations. Ion mobility mass spectrometry (IM‐MS) experiments by Dupuis et al.^16^ determined collisional cross sections that suggested that rIAPP has two dominant conformations. When characterized by molecular dynamics (MD) simulations, these conformations were shown to be: (1) mostly turn and coil secondary structure and a helix‐coil conformation and, (2) a structure containing a short turn‐coil (residues Lys1–Cys7), a short helix (residues Ala8–Val17), and a long turn‐coil (residues Arg18–Tyr37).

Similar studies with hIAPP under physiological solution conditions have not been possible because of its high tendency for aggregation. An NMR study investigated the monomeric form of the free acid form of hIAPP (with an additional Gly at the C‐terminus) as it has a lower propensity for aggregation. This study found that the N‐terminal region has a tendency for transient helical conformations; however, long‐range resonances were not observed, indicating that the peptide does not adopt a unique 3D structure or fold.^17^ This suggests that hIAPP has very similar Cα chemical shifts to rIAPP, with residues Lys1–Ser20 exhibiting transient helical propensity and a significantly lower structural propensity in residues Asn21–Tyr37.

Table 1 shows the NMR structural ensemble of rIAPP obtained in dodecylphosphocholine micelles, which reveals that in such a lipidic environment, rIAPP contains an α‐helical region at residues Ala5‐Leu23.^18^ The C‐terminal region is disordered as the presence of Pro 25, 28, and 29 prevents the formation of a compact α‐helical structure. Table 1 also shows the corresponding NMR structural ensemble of hIAPP in micelles,^19^ which reveals that in such an environment, hIAPP exhibits extensive α‐helical structure. It has been proposed that the lack of α‐helical structure at the C‐terminal region of rIAPP compared with hIAPP limits the ability of rIAPP to aggregate into insoluble fibrils.^6^, ^19^, ^20^ However, the limited structural information available for rIAPP in solution under physiological conditions due to its intrinsically disordered nature has hampered understanding of the mechanism of aggregation of IAPP.

MD simulation methods, if appropriately validated with experimental data, can provide structural information about IDPs that is difficult to obtain experimentally, such as is indeed the case for the monomeric forms of rIAPP and hIAPP in solution. However, the accurate prediction of proteins' structural properties by MD simulations relies on two important factors: using an appropriate force field that can accurately represent the propensity for different secondary structures and a suitable method to sample their conformational landscape.

Several MD simulation studies have focused on characterizing the solution structures of hIAPP and rIAPP. The first simulation of full‐length IAPP was reported by Dupuis et al.^16^ using replica exchange MD (REMD) in the gas phase and in implicit aqueous solvent. This study determined the β‐sheet and α‐helical propensities of hIAPP (36% and 6%, respectively) and rIAPP (7% and 36%, respectively). In both hIAPP and rIAPP, the N‐terminal region (residues Lys1–Ala/Pro25) exhibited a propensity to form α‐helical structures, but to a different extent. It is thought that the loss of this α‐helical propensity and the formation of β‐sheet structure in the N‐terminal region of hIAPP causes aggregation.^21^ Reddy et al.^15^, ^22^ used REMD and replica exchange with umbrella sampling to characterize the monomeric forms of both rIAPP and hIAPP in solution. hIAPP frequented three stable conformations: (1) a compact α‐helical/coil structure with the α‐helical segment comprised of residues Thr9–Val17, with a short antiparallel β‐sheet comprising residues Gly24–Ser28 and Asn31–Asn35; (2) an extended anti‐parallel β‐sheet structure with a turn conformation in residues Ser20–Phe23; and (3) a completely unstructured (random coil) conformation. By contrast, rIAPP frequented only two dominant conformations: (1) a compact α‐helical conformation comprising residues Cys7–Val17, consistent with the NMR structure of rIAPP in a micellar environment; and (2) an extended random coil conformation. Hoffmann et al.^11^ used bias exchange metadynamics (BEMD) to study hIAPP and rIAPP in solution while testing six different force fields in combination with various water models, namely AMBERff99SB*‐ILDN with TIP3P/TIP4P, AMBERff03w with TIP4P/TIP4P2005, CHARMM22/CMAP with TIPs3P, CHARMM22* with TIPS3P/TIP4P, GROMOS96 53a6 with SPC, and OPLS‐AA/L with TIP4P. The conformational free energy landscapes of both hIAPP and rIAPP were mostly similar, with the lowest free energy regions corresponding largely to random coil structures. However, hIAPP was determined to more readily adopt structures containing transient α‐helices and β‐strands than rIAPP. Comparison with NMR chemical shift data for rIAPP in solution suggested that AMBERff99SB*‐ILDN with TIP4P, AMBERff03w with TIP4P2005, and CHARMM22* with TIP4P were the force field and water model combinations that best represented the balance of secondary structures of rIAPP in solution, a conclusion which was also extrapolated to hIAPP. Peng et al.^23^ tested five different force fields in combination with various water models (AMBERff99SB*‐ILDN with TIP3P, CHARMM36 with TIPs3P, CHARMM22* with TIP3P/TIPs3P, and CHARMM27 with TIP3P/TIPs3P, and GROMOS 54a7 with SPC) for the prediction of the conformational free energy landscape of hIAPP. Both conventional (unbiased) MD (cMD) and replica exchange with solute tempering (REST2) were used. AMBERff99SB*‐ILDN with TIP3P and CHARMM22* with TIP3P were stated as showing the best agreement with experiment; however, this comparison was made to the NMR and CD spectroscopy data for fragments taken from the fibrillar form of hIAPP.^24^ A detailed comparison of MD simulation studies of IAPP was recently discussed by Moore et al.^25^

In this work, the conformational ensembles of rIAPP and hIAPP were predicted using well‐tempered bias exchange metadynamics,^26^, ^27^ to use an enhanced sampling method that can comprehensively describe the conformational free energy landscape of these peptides. To test several newly developed force fields, six popular force field/water model combinations were compared and validated using NMR chemical shift data for rIAPP in solution. The most appropriate force fields for representing the conformational preferences of rIAPP were identified, and one was used to simulate hIAPP. This then facilitated the characterization of the structural differences between rIAPP and hIAPP to help to establish a structure–function relationship for the aggregation properties of hIAPP.

## 2 METHODS

### 2.1 Preparation of initial structures

To obtain equilibrated structures of rIAPP and hIAPP as starting conformations for the subsequent metadynamics simulations, a cMD simulation with each force field was conducted for 40 ns. Monomers of rIAPP or hIAPP in a fully extended conformation were prepared using Discovery Studio (Biovia, Dassault Systèmes). For all simulations, the peptides were modeled with the N‐terminus capped with an NH_3_^+^ and the C‐terminus capped with an amidated (NH_2_) group for consistency with experimental studies. The net charge of rIAPP was +4 and of hIAPP was +3. The peptide was placed into a cubic simulation cell with a minimum distance of 1.0 nm to the edge of the cell and solvated with ~7700 water molecules. To neutralize the charges of the peptides, four and three Cl^−^ ions were added to rIAPP and hIAPP, respectively, and additional ions (22 Na + and 22 Cl^−^) were added to reach a physiological ionic strength of ~150 mM. From each simulation, a structure was extracted whereby the Cα of Cys2 and Cys7 were within ~0.3 nm to allow for the formation of a disulfide bond. These structures were used to prepare new simulation systems in which the peptide had the required disulfide bond between Cys2 and Cys7. The peptide was solvated, and ions were added as before. Each system was energy minimized, followed by a simulation for 100 ps in the NVT ensemble (at a density of 0.999 g/cm^3^) and for 20 ns in the NPT ensemble for equilibration. All previously described cMD simulations were conducted using the Gromacs 5.0.7 package^28^ with the following conditions for all force fields. The temperature was kept at 310 K using the Nosé‐Hoover thermostat with a time constant of 2.0 ps.^29^, ^30^ The pressure was held at 1 atm with the Parrinello and Rahman^31^ barostat with a time constant of 6.0 ps and compressibility of 4.5 × 10^−5^ bar^−1^.^32^ Electrostatic interactions were computed using the particle mesh Ewald method^33^, ^34^ with a *r*_coulomb_ cutoff of 0.9 nm. A twin range cutoff scheme was used for the van der Waals interactions with a *r*_vdW_ cutoff of 0.9 nm. Periodic boundary conditions were applied in all directions, and a time step of 2 fs was used throughout.

### 2.2 Combinations of force fields and water models

The six force fields/water models tested with rIAPP are listed in Table 2 and the best was chosen to simulate hIAPP. The selection of force fields was chosen as a mix of the different families (CHARMM, Amber, GROMOS, and OPLS) and a mix of force fields previously tested with IAPP and newly developed ones. Similar methods and collective variables (CVs) allowed comparison with work previously done by Hoffmann et al. on rIAPP.^11^ We chose two force field/water model that were tested by Hoffmann et al. as a direct comparison and validation to our work. They indicated AMBERff99SB*‐ILDN and CHARMM22* as their most accurate force fields, so we replicated this with the updated water model TIP4Pd. Their work showed the limitations of Gromos 53a6, so we tested the updated Gromos 54a7. One newly developed force field, specifically tested on an amyloidogenic IDP, Amberff99SBnmr2 with TIP4Pd, was added as a comparison to those force fields previously seen to be accurate for hIAPP. There are indeed other more recently developed force fields aimed at IDPs, which future studies could expand into.

**TABLE 2:** List of protein force fields and water models tested

Force field	Water model
AMBERff03w^35^	TIP4P/2005^36^
AMBERff99SB*‐ILDN^37^, ^38^, ^39^, ^40^	TIP4Pd^41^
AMBERff99SBnmr2^42^	TIP4Pd^41^
CHARMM22*^43^	TIP4P^44^
GROMOS 54a7^45^	SPC^46^
OPLS‐AA/L^47^	TIP4P^44^

## 3 METADYNAMICS SIMULATIONS

MD simulations using well‐tempered bias exchange metadynamics^26^, ^27^ were conducted using the Plumed 2.3.5 plugin^48^ with Gromacs 5.0.7.^49^ Metadynamics is an enhanced sampling method that uses a history‐depended bias potential (a Gaussian energy function or “hill”) to prevent systems from continually sampling the same configurations, thus driving the system into high‐energy configurations that may not be as favorable but that contribute to the free energy of the system.^50^ Well tempering prevents oversampling the free energy landscape by decreasing the height of the Gaussian functions added.^26^ Two CVs, α‐root mean square deviation (RMSD) and antiparallel β‐RMSD^51^ were used to bias sampling of α‐helical and β‐sheet conformations. These CVs use a slider to calculate the RMSD of multiple segments of the peptide with respect to an ideal α‐helical or β‐sheet structure (six consecutive residues for α‐RMSD and three + three residues for β‐RMSD), which is then summed to provide a measure of total α‐helical or β‐sheet content. Simulations were run with a BEMD scheme of three replicas: one biased along α‐RMSD, one biased along β‐RMSD, and one biased on both α‐RMSD and β‐RMSD. A wall was placed at α‐RMSD 20 and β‐RMSD 8 to restrict sampling to the region of interest. The parameters used for the switching function in the RMSD CVs were: for α‐RMSD *r*0 = 0.1, *M* = 8, and *N* = 4, and for β‐RMSD *r*0 = 0.1, *M* = 12, and *N* = 6.^52^, ^53^ A Gaussian hill (bias potential) of height 2.0 kJ/mol and sigma of 0.1 with a well‐tempering bias factor of 15 was deposited every 500 steps, and an exchange was attempted every 120 ps, with an exchange frequency of ~20% with all force fields. Trajectory frames were saved every 100 ps for subsequent analysis.

The free energy of each conformation was computed as a function of both CVs using the METAGUI plugin in VMD.^54^ Each of the simulations was conducted for 1–1.3 μs (for each replica), with convergence of the free energy reached between 400 and 800 ns (per replica) in each system. The free energy was computed using all frames in the simulations, and all other analysis was performed using only the postconvergence region of the trajectories. Figure S1 reports analyses of the changes in the free energy landscape as a function of time, which were used to assess convergence.

All properties extracted from the simulations were reweighed using the method described in Zerze et al., which is equivalent to other reported reweighting methods.^55^, ^56^, ^57^ METAGUI calculates the free energy for each microstate, and the following formula can be applied to each observable property postconvergence.

$$\;\left\langle O\right\rangle =\frac{\sum_{\alpha }O_{\alpha }e^{-F_{\alpha }/T}}{\sum_{\alpha }e^{-F_{\alpha }/T}}$$

Where the sum is across all the microstates and *O*_α_ is the arithmetic average of the observable property across all configurations within microstate *α*.^55^ This approach computed average chemical shifts and secondary structure properties using in‐house scripts.^58^

## 4 NMR SECONDARY CHEMICAL SHIFT CALCULATIONS

In each one of the simulations, structures of rIAPP and hIAPP were extracted every 20 ps after convergence had been reached, and their NMR Cα chemical shifts were calculated using the program SHIFTX2.^59^

The chemical shift values of each frame were reweighted as described above, after which sequence‐corrected random coil values^60^, ^61^ were subtracted to obtain secondary chemical shift values. Predicted secondary Cα chemical shifts were compared with the corresponding NMR experimental values reported by Williamson and Miranker^14^ for rIAPP in solution. The primary experimental chemical shift data also had sequence‐corrected random coil values subtracted to enable direct comparison. It should be noted that experimental chemical shift values were obtained at 278 K, whereas our chemical shifts were predicted at 310 K. However, the effect of the different temperatures on the predicted values of chemical shifts was assessed by Hoffmann et al.^11^ and no statistically significant differences were observed.

## 5 SECONDARY STRUCTURE ANALYSIS

Define secondary structure of proteins (DSSP) analysis was performed using the Gromacs module do_dssp^62^, ^63^ on all time frames post‐convergence and reweighted as described above. The output was grouped into four categories: unordered or random coil (coil, bend, beta‐bridge, and π‐helix), turn, β‐sheet, and helix (α‐helix and 3–10 helix)^64^ to describe the percentage and fraction secondary structure of each amino acid residue in the peptide sequence.

## 6 RESULTS

### 6.1 NMR secondary chemical shifts

**FIGURE 1:**
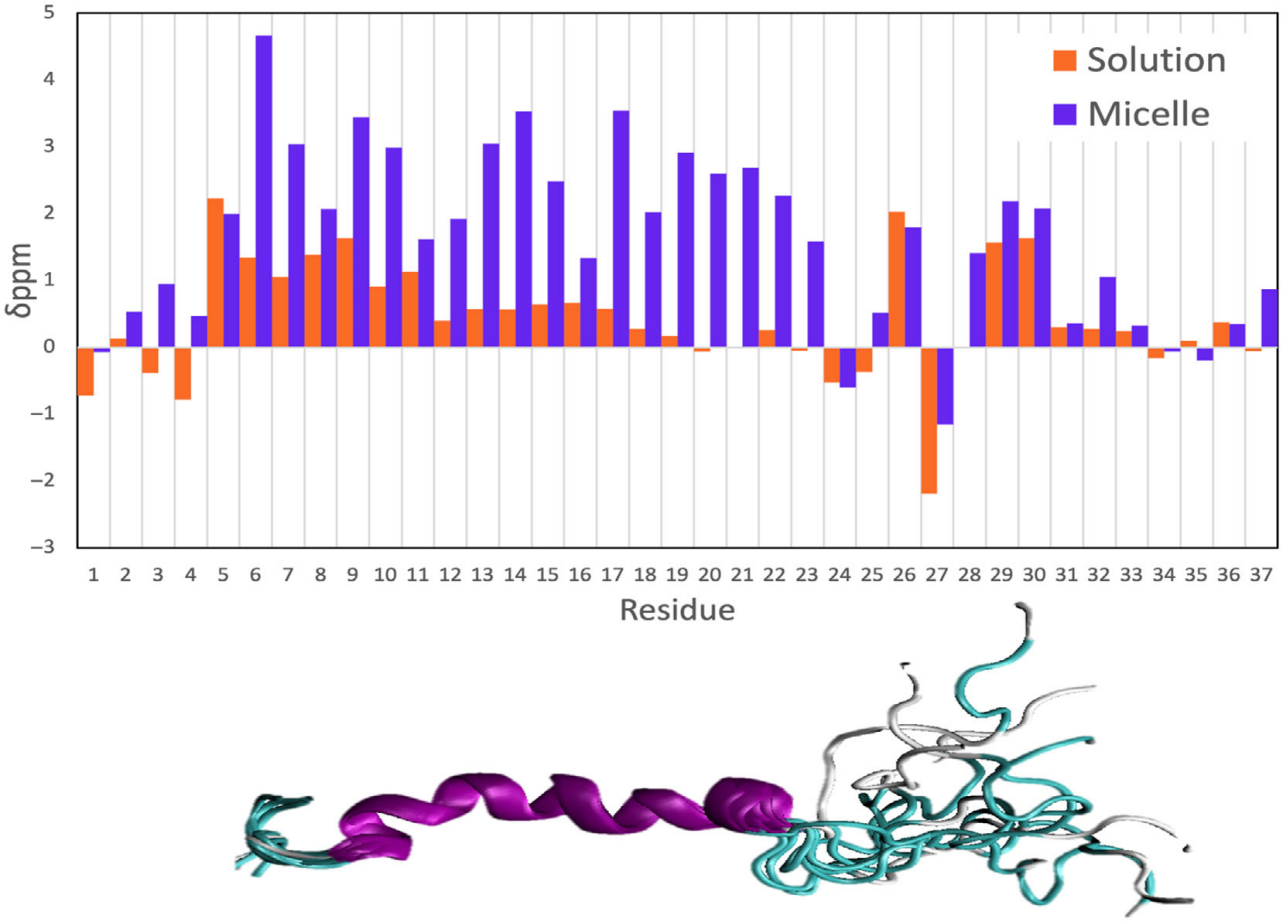
Experimental Cα secondary chemical shifts of rat islet amyloid polypeptide in solution and a micelle environment. The corresponding structural ensemble in the micelle is shown in ribbon representation, with the α‐helical region colored in purple.

Comparison of predicted and experimental Cα secondary chemical shifts can be used to validate the conformational ensemble predicted by MD simulations and provide an indicative measure of average secondary structure propensity on a per residue basis. Accordingly, Cα secondary chemical shifts predicted from the simulations conducted with each force field were compared with the experimental values in aqueous solution reported by Williamson and Miranker.^14^ Values greater than 0 indicate an average α‐helical propensity, near 0 are an average propensity for random coil conformations, and values less than 0 are an average β‐sheet propensity.^65^ Secondary chemical shifts were calculated as an average over conformations sampled after convergence by subtracting standard random coil values. The values of the secondary chemical shifts reported are reweighted averages over all conformations sampled and, like with experimental values, reflect the transient nature of the present secondary structure. Consequently, they may not directly reflect the low energy of random coil conformations with limited secondary structure, as described by the free energy landscapes. Figure 1 compares the experimental Cα secondary chemical shifts for rIAPP in solution and a micelle environment.^18^ Despite exhibiting similar upfield chemical shifts, it is evident that the NMR structural ensemble exhibits much larger α‐helical (upfield) shifts, which reflect the dominant and extensive nature of the observed α‐helical structure of rIAPP when bound to a micelle.^18^ This reveals that the values of the secondary chemical shifts for rIAPP in solution are relatively small in magnitude and do not reflect dominant (i.e., long‐lived) and substantial preferences in secondary structure. For comparison, a fully formed, stable α‐helix would exhibit values of Cα secondary chemical shifts >2.6 δ ppm over multiple residues.^14^, ^66^, ^67^

## 7 COMPARISON OF FORCE FIELDS FOR rIAPP

Experimental NMR chemical shifts for rIAPP indicate the presence of a transient helix in the N‐terminal region for residues Ala5‐Ser19.^14^ This N‐terminal region has also been shown to have a long‐lived helical conformation in a micelle environment (Table 1).^18^ Previous MD simulation studies have also reported an α‐helical tendency for the N‐terminal region of rIAPP, with Dupuis et al.^16^ predicting that residues Lys1–Pro25 exhibit α‐helical conformations and Reddy et al.^15^ predicting that residues Thr9–Val17 have α‐helical conformations. This N‐terminal region of the peptide contains several residues that in globular proteins are known to have large α‐helical propensity (Ala, Gln, and Leu).^68^ In contrast, the C‐terminal region has previously been shown to be disordered, with NMR, IM‐MS, and MD studies showing an unstructured coil‐turn conformation for residues Arg18‐Tyr37.^14^, ^15^, ^16^

**FIGURE 2:**
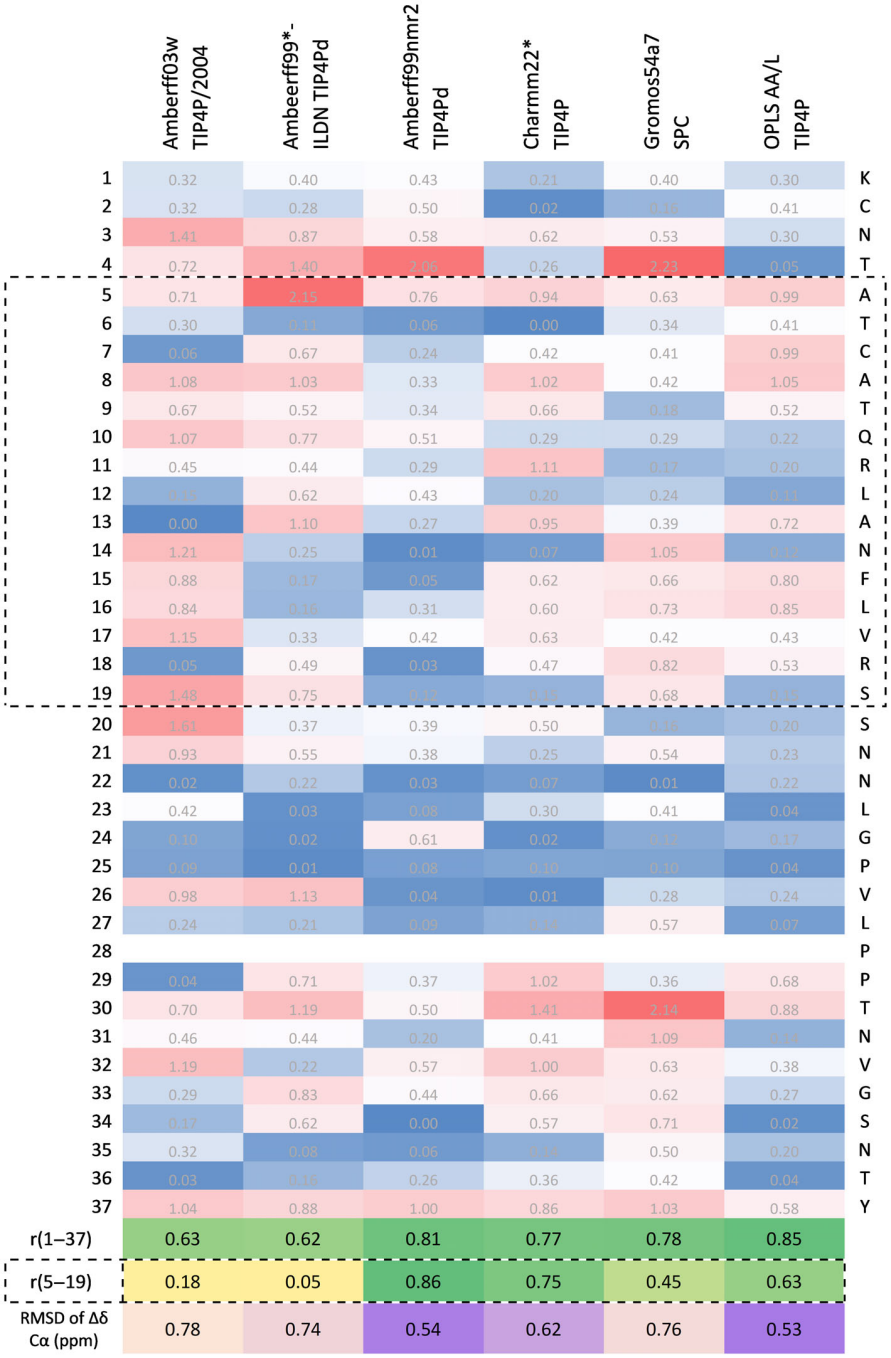
Heat map of the difference between experimental and predicted Cα secondary chemical shift values for each residue and each force field. Blue indicates the most accurate predictions, whereas red indicates the least accurate. The dashed lines indicate residues with known α‐helical propensity. R(1–37) report the Pearson's correlation coefficient for the entire peptide and r (5–19) for the 5–19 region, with yellow highlighting the lowest correlation and darkest green highlighting the highest correlation. Root mean square deviation (RMSD) of Δδ Cα (ppm) is a measure of similarity between experiment and predicted chemical shift values, with purple color being used to highlight values more similar to experiment and orange color indicating the least similar to experiment.

**FIGURE 3:**
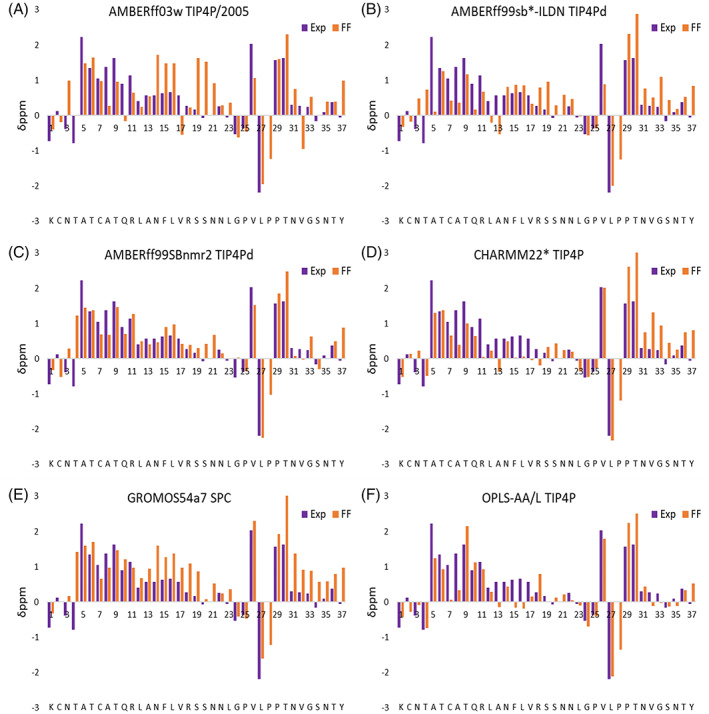
Predicted Cα secondary chemical shift values for each residue and for each force field/water model combination for rat islet amyloid polypeptide using the SHIFTX2 program, with upfield shifts indicating α‐helical trends and downfield shifts indicating β‐sheet trends. The average standard error for all force fields per residue was calculated to be 0.012.

To assess the accuracy of each force field, the corresponding predicted Cα secondary chemical shifts were compared with the experimental values shown in Figures 2 and 3 (Table S1 reports the correlation with experimental values of the predicted secondary chemical shifts for the other mainchain atoms). Figure 2 is a heat map that illustrates the accuracy of the predicted secondary chemical shifts for each force field with respect to experimental values for each residue. The associated Pearson's correlation coefficient was calculated for each force field/water model combination. These values are shown at the bottom of Figure 2, with the highest value reflecting the highest accuracy. The second set of Pearson's correlation coefficients was calculated for the secondary chemical shift values of residues Ala5–Ser19 (the region highlighted by a dashed square in Figure 2). The RMSD of Δδ ppm Cα for each force field/water model combination was also calculated and appears at the bottom of Figure 2. This is another measure of similarity between predicted and experimental secondary chemical shifts, with the lowest value (highlighted in purple) indicating the most accurate predictions. Figure 3 shows the predicted Cα secondary chemical shifts for each force field/water model combination and the corresponding experimental values for each residue. Table S2 shows that all force fields predict rIAPP to have similar percentage content of secondary structure as calculated by DSSP.

Figure 2 shows that the force field with the highest Pearson's coefficient and lowest RMSD of Δδ ppm Cα was OPLS‐AA/L with TIP4P, which exhibited, on average, the best agreement with experimental data throughout the entire peptide sequence (*r* = .85). However, the per residue Cα secondary chemical shifts in Figure 3 shows that the force field underestimates the α‐helical propensity for residues Arg11–Val17, resulting in a lower correlation coefficient for the central region. The correlation coefficient further shows that AMBERff99SBnmr2/TIP4Pd has the next best agreement across the entire peptide and the best agreement for the central region of interest (Ala5–Ser19). Figure 3 shows that this force field has the best agreement across the N‐terminal half of the peptide and does not overestimate the α‐helical propensity at the C‐terminal, unlike most other force fields. AMBERff99SBnmr2/TIP4Pd shows a low value of RMSD of Δδ ppm Cα with 0.54 ppm. This value is similar to the 0.442 and 0.509 ppm values reported by Song et al.^69^ for two newly developed force fields (AMBERff14IDPSFF and AMBERESFF1 with TIP3P) specifically parameterized for IDPs. The next two force fields in the ranking are CHARMM22*/TIP4P and GROMOS54a7/SPC, both of which show overestimation of the α‐helical propensity at the C‐terminal region. GROMOS54a7/SPC also overestimates the α‐helical propensity of the middle of the peptide, whereas CHARMM22*/TIP4P underestimates the α‐helical propensity of this region. Next, the AMBERff03w/TIP4P overestimates the α‐helical propensity of the middle region of the peptide (residues 14–21) whilst accurately predicting the α‐helical trend at the N‐terminal region. AMBERff99SB*‐ILDN/TIP4Pd underestimates the α‐helical propensity at the N‐terminal but overestimates the α‐helical trend at the C‐terminal region, which is also seen in other force fields. Overall, most force fields slightly over predict the helical propensity of regions Asn14–Leu23 and Pro29–Tyr37, whilst accurately reflecting the helical trend observed experimentally for residues Lys1–Leu12 and the large fluctuations observed for residues Gly24–Pro28.

**FIGURE 4:**
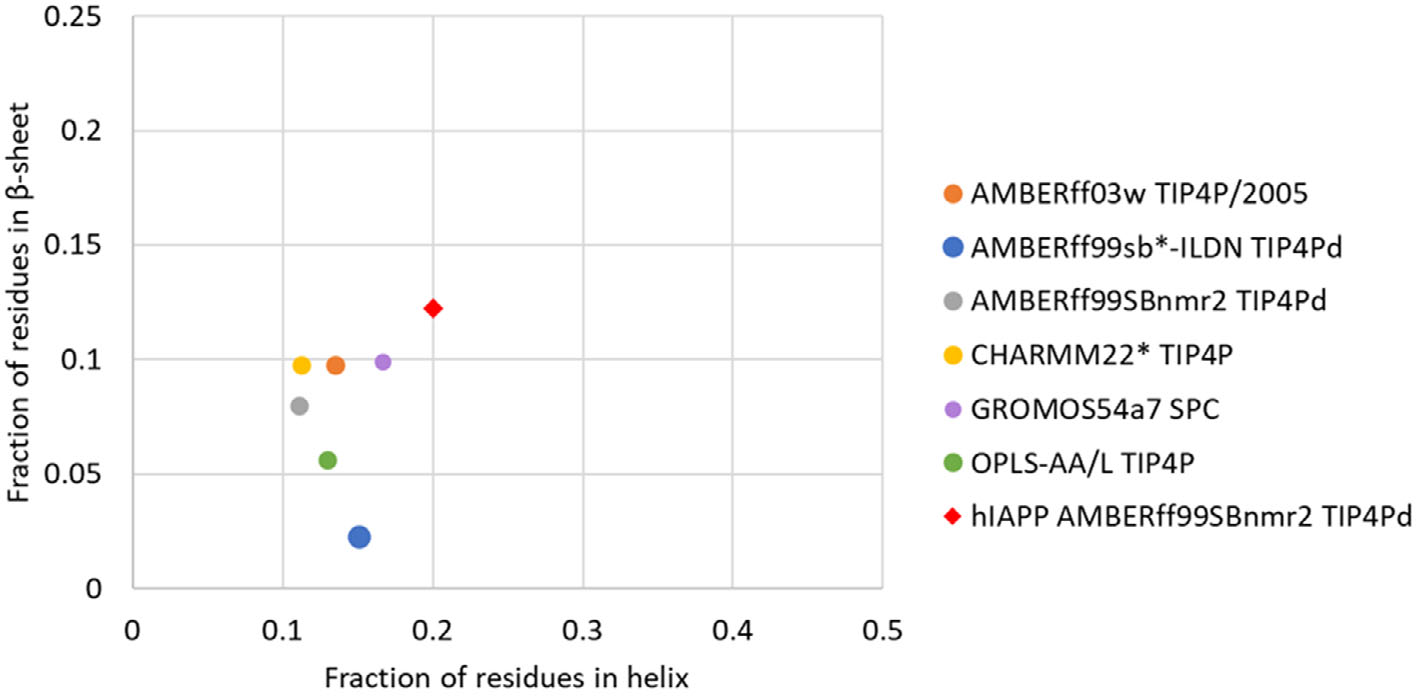
Comparison of the fraction of residues in helical or β‐sheet conformations. Circles represent rat islet amyloid polypeptide in different force fields, and the diamond corresponds to human islet amyloid polypeptide (hIAPP).

A comparison of the fraction of residues observed in each secondary structure is reported in Figure 4. Only a low proportion of residues indicate a preference for helical or β‐sheet conformations, with all force fields predicting <0.2 in both conformations for rIAPP. AMBERff99SBnmr2 with TIP4Pd is shown to predict the lowest fraction of residues in β‐sheet conformations. AMBERff99SBnmr2 with TIP4Pd and OPLS‐AA/L with TIP4P accurately predicted the experimental Cα secondary chemicals values and resulted in the lowest predicted fraction of residues in β‐sheet conformation for rIAPP. This figure can be directly compared with the data reported by Hoffmann et al.,^11^ who reported a similar analysis with some of the force fields included in our study. OPLS‐AA/L is predicted to have a similar fraction of residues with secondary structure, as reported by Hoffmann et al, with around 0.12 helical and 0.05 β‐sheet. AMBERff03w with TIP4P/2005 and CHARMM22* with TIP4P predict similar helical values to Hoffmann et al. (1.1–1.3 for α‐helix); however, a slightly higher β‐sheet fraction is predicted (0.05–0.09).

## 8 COMPARISON OF CONFORMATIONAL PREFERENCES IN rIAPP AND hIAPP

**FIGURE 5:**
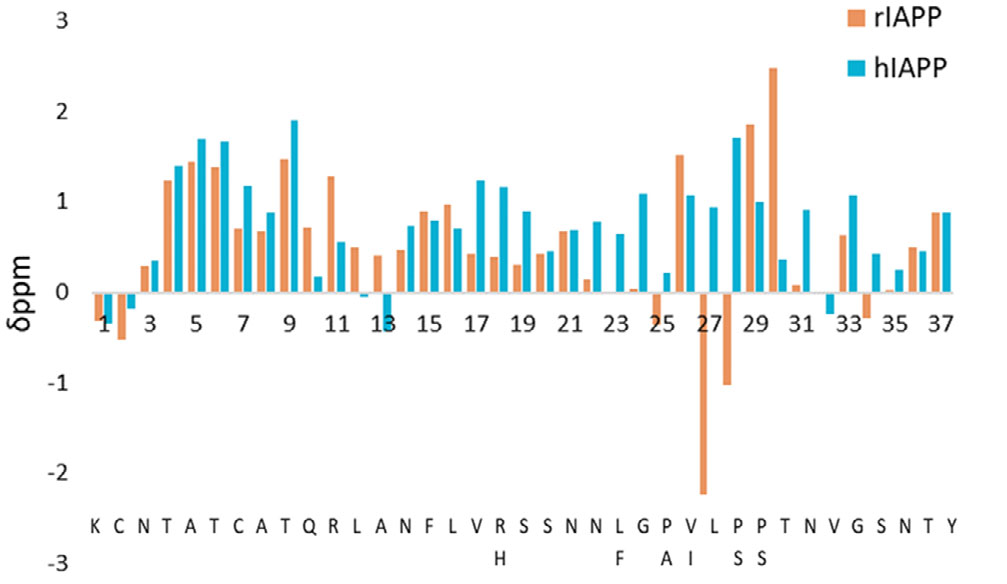
Predicted Cα secondary chemical shift values for each residue of rat islet amyloid polypeptide (rIAPP) and human IAPP (hIAPP) using the AMBERff99SBnmr2/TIP4Pd force field and water model combination.

As previously discussed, the disordered nature of rIAPP and hIAPP has made it difficult to characterize their structures experimentally, with the added difficulty of their tendency to aggregate, particularly for hIAPP. To further understand and contrast the structural preferences of these peptides, the best‐performing force field was chosen for simulating hIAPP: AMBERff99SBnmr2/TIP4Pd. Figure 5 shows the values of the Cα secondary chemical shifts predicted using Shiftx2 for both rIAPP and hIAPP, and Figure 5 shows the percentage secondary structure of each amino acid residue for both peptides determined using DSSP.

As discussed above, experimental NMR studies in solution and a micelle environment have indicated that rIAPP has a transient α‐helical region at residues Ala5–Ser19. The simulations of hIAPP and rIAPP both predict the presence of α‐helical propensity in the N‐terminal region, where they share 100% sequence identity (Lys1–Val17). hIAPP is also predicted to have an α‐helical propensity in its C‐terminal region (Ile26–Tyr37). The secondary structure % (Table 3) confirms this as rIAPP is predicted to exhibit higher random coil content than hIAPP, whereas hIAPP is predicted to have increased helical content, also observed in Figure 4. This is consistent with the experimental structures reported in Table 1, with the α‐helical conformation in the C‐terminal region of hIAPP prevented in rIAPP by the Pro residues present in its sequence and other simulations studies.^70^ The free acid form of hIAPP was shown experimentally to have very similar Cα chemical shift values to those of rIAPP for residues Lys1–Ser20, indicating the presence of a similar transient helical structure.^17^

**TABLE 3:** Secondary structure percentage content in rIAPP and hIAPP calculated using DSSP

	rIAPP	hIAPP
Coil	67.2	52.4
Turn	13.8	15.3
Helix	11.0	20.0
β‐sheet	8.0	12.2

Abbreviations: DSSP, define secondary structure of proteins; hIAPP, human islet amyloid polypeptide; rIAPP, rat islet amyloid polypeptide.

**FIGURE 6:**
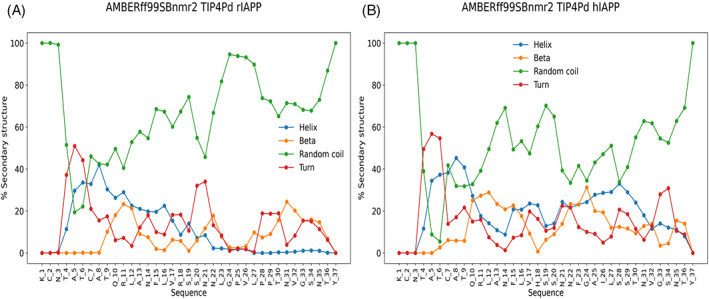
Secondary structure percentage content per residue in rat islet amyloid polypeptide (rIAPP) and human IAPP (hIAPP) determined by define secondary structure of proteins. Random coil (coil, bend, β‐bridge and 5–10 helix), turn, β‐sheet, and helix (α‐helix and 3–10 helix)

Of particular interest are the residues that differ between hIAPP and rIAPP: His18Arg, Phe23Leu, Ala25Pro, Ile26Val, Ser28Pro, and Ser29Pro. Residue 18 in both Figures 5 and 6 shows a larger helical content in hIAPP: Phe (60%) compared with His (8%) in rIAPP. Furthermore, neighboring residues Val17 and Ser19 also exhibit higher helical content in hIAPP than in rIAPP. Leu23 is predicted to be the endpoint of the helical propensity in rIAPP, whereas Phe23 in hIAPP shows significant helical propensity, as indeed do the contiguous residues Asn22 and Gly24. Recent mutagenesis studies have shown that mutations of Phe23 and Ile26 to alanine significantly decrease the self‐aggregation tendency of hIAPP and its ability to co‐aggregate with amyloid β.^71^ The other residues of interest are 25, 28, and 29, which are proline in rIAPP and have been shown experimentally to be a reason for rIAPP aggregating to a much lesser extent than hIAPP.^9^ Pramlintide is a mutated peptide form of hIAPP with the three proline substitutions from rIAPP and which has been shown to have reduced aggregation tendency and can inhibit the formation of long β‐sheet conformations.^72^ The chemical shifts (Figures 3 and 5) reveal that residues Leu27 and Pro28 in rIAPP are predicted by all force fields to exhibit substantial downfield Cα secondary chemical shifts. This effect is not predicted for Leu27 and Ser28 in hIAPP. In Figure 6, DSSP analysis shows that the three proline residues 25, 28, and 29 have a much higher random coil percentage content (93%, 89%, and 73%, respectively) than the corresponding residues Ala25, Ser28, and Ser29 in hIAPP (43%, 51%, and 33%, respectively), which reveal a decrease in the tendency to adopt α‐helical conformation. The three proline residues in the C‐terminal region of rIAPP can thus hinder the α‐helical tendency observed in hIAPP across these residues. This is consistent with experimental Cα secondary chemical shifts for rIAPP in solution^14^ and the NMR structural ensemble observed in a micelle environment^18^ (Table 1 and Figure 1), which show that hIAPP has a propensity for α‐helical conformation at the C‐terminus, which is not observed in rIAPP.

The central region of hIAPP (Ser20–Pro29) is another region of interest when comparing the conformational preferences of hIAPP and rIAPP. This region is the smallest fragment of hIAPP that can affect the formation of β‐sheet fibrils.^73^, ^74^, ^75^ Along with the C‐terminus, this region is predicted to have a higher α‐helical propensity in hIAPP. This is again consistent with the experimental micelle structures of hIAPP and rIAPP, where an α‐helical structure is observed in hIAPP but not in rIAPP.^18^, ^19^ It is important to note that this central region in hIAPP also contains residues usually found at the edge of β‐strands to allow the formation of bends (Pro and Gly).^76^

## 9 CONFORMATIONAL FREE ENERGY LANDSCAPES

**FIGURE 7:**
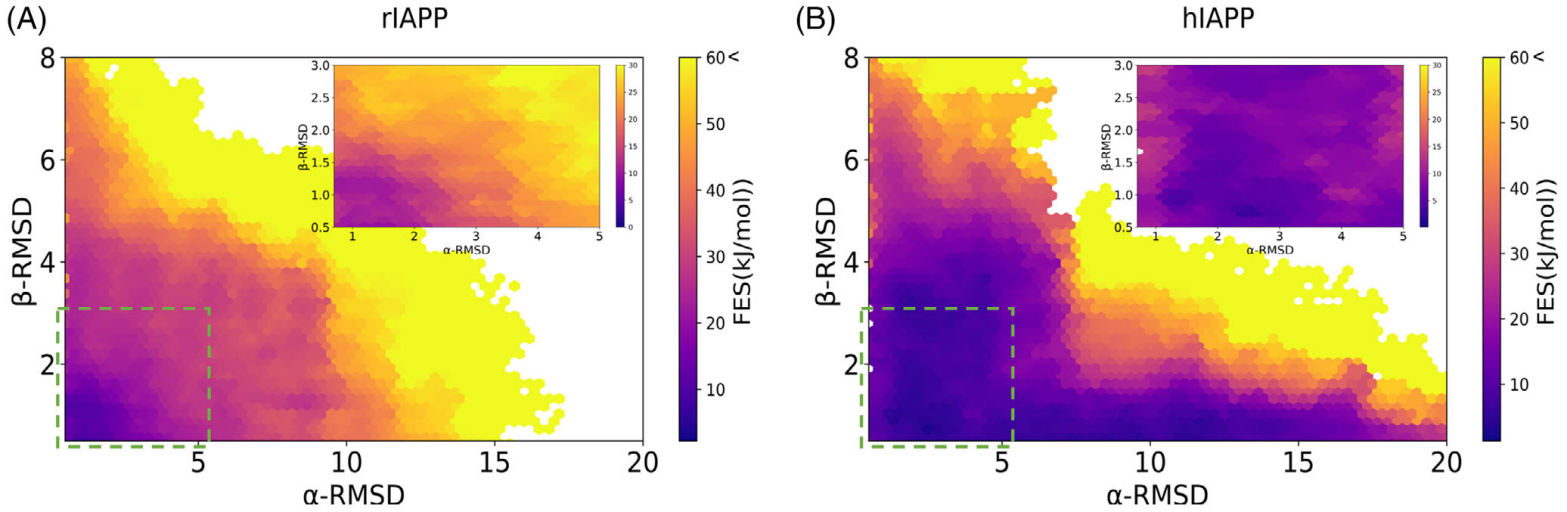
Conformational free energy landscapes of rat islet amyloid polypeptide (rIAPP) and human IAPP (hIAPP) for the AMBERff99SBnmr2/TIP4Pd force field/water model combination. Free energies are shown as a function of two collective variables: α‐root mean square deviation (RMSD) on the x‐axis and β‐RMSD on the y‐axis. The darker purple regions indicate lower free energy. The inset focuses on the area in the green square, with the free energy range reduced to 30 kJ/mol.

The conformational free energy landscapes of rIAPP and hIAPP in solution predicted using the best force field/water model combination are shown in Figure 7 (Figure S2 shows the free energy landscapes of rIAPP for the other five force field/water model combinations). These free energy surfaces are shown as a function of the two CVs used, α‐RMSD and β‐RMSD, which reflect the α‐helical and antiparallel β‐sheet structural characteristics of the entire peptide. The values of α‐RMSD do not reflect other types of helical conformations (e.g., helical turns, 3_10_ helices, or π‐helices), whereas the values of β‐RMSD do not reflect parallel β‐sheet conformations. Consequently, the values of these CVs will underestimate these other specific conformations (although the presence of parallel β‐sheet content in short proteins is not expected). Equally, these values also do not unambiguously define secondary structure because the location and extent of regions with specific secondary structure cannot be distinguished. For example, high values of α‐RMSD indicate that many residues exhibit α‐helical conformation but do not inform if these residues are contiguous and thus form a long α‐helical stretch or if this reflects the existence of several shorter α‐helical stretches.

The region of most interest in the conformational free energy landscape corresponds to areas with the lowest free energy (i.e., the darkest purple/black parts in Figure 7). There is some degree of variability between force fields/water model combinations, as revealed by the predicted free energy surfaces (Figure S2). Most force fields exhibit a common low free energy region around CV values of 1 in α‐RMSD and 2 in β‐RMSD^11^; however, the size and location of other low free energy regions are different for all force fields, which likely reflect differences in how each force field is parameterized as well as the water model.

Both rIAPP and hIAPP have their lowest free energy state in low values of α‐RMSD (<2), with the darkest purple regions in Figure 7 being close to the origin of the x‐axis and y‐axis. The inset within Figure 7 focuses on the low CV range, showing that hIAPP has conformational free energies within 10 kJ/mol over the ranges of 0–5 α‐RMSD and 0–3 β‐RMSD, whereas the corresponding low free energy range in rIAPP extends only to below values of 2 α‐RMSD and 1.5 β‐RMSD. A comparison of the energy landscapes shows that hIAPP has a flatter energy landscape compared with rIAPP, with a larger dark purple region indicating a larger range of CV values with low free energy. hIAPP is also predicted to reach higher CV values with much lower free energies than rIAPP. Conformations with the highest free energy in rIAPP were reached for values around 17 for α‐RMSD, whereas equivalent free energies were reached in hIAPP at values of around 20 for α‐RMSD. This trend is consistent with findings from NMR, IM‐MS, and previous MD simulation studies that indicate that hIAPP can exhibit more α‐helical structure in its C‐terminal region compared with rIAPP due to the absence of Pro residues.

## 10 DISCUSSION

This work aimed to assess the influence of the force field/water model combination on the predicted conformational free energy landscape of rIAPP predicted by BEMD simulations and to provide a comprehensive comparative analysis of the conformational free energy landscapes of rIAPP and hIAPP. Force fields for peptides and proteins tend to exhibit biases in the prediction of secondary structure in IDPs and therefore, assessment of force fields with IAPP is imperative. In this study, six force field/water model combinations were investigated to assess the accuracy of predictions of experimental secondary chemical shifts.

The AMBERff99SBnmr2^42^ force field, combined with the TIP4Pd water model, was the most accurate at predicting the experimental NMR secondary chemical shifts of rIAPP. The RMSD of Δδ ppm Cα indicates similar accuracy as the IDP‐specific force fields AMBERff14IDPSFF and AMBERESFF1.^69^ This force field was recently developed to improve the conformational ensemble of IDPs and disordered regions in proteins using the TIP4Pd water model, also optimized for IDPs. Backbone dihedral angle potentials were rebalanced in a residue‐specific manner to quantitatively reproduce dihedral angles of random coils. This force field was parameterized with amyloid‐β and α‐synuclein (other amyloidogenic IDPs), showing good prediction of backbone conformation ensembles; however, there has been limited evaluation with proteins or other IDPs.^42^, ^77^ The TIP4Pd^41^ water model was developed to correct the underestimation of London dispersion interactions present in other water models, enabling a better representation of conformational ensembles of IDPs.^41^ Use of this water model has been shown to better reproduce the radius of gyration and NMR observables in IDPs than TIP3P.^69^ Our findings indicate that this force field, along with the TIP4Pd water model, is accurate at predicting experimental properties of IAPP.

The OPLS‐AA/L^47^, ^78^ force field, combined with the TIP4P water model, was the second most accurate at predicting the secondary chemical shifts of rIAPP compared with experimental values. This force field, released in 2001, was reparameterized for better representation of peptide folding by refitting the backbone torsional coefficients using the TIP4P water model.

CHARMM22*^43^ is a reparameterization of CHARMM22 that improves the helix‐coil balance by modifying the backbone torsional potentials and which is effective for the simulation of the folding of proteins. Unsurprisingly, this force field, combined with the TIP4P water model, was fairly accurate at predicting the Cα secondary chemical shifts of rIAPP, confirming previous similar predictions.^11^, ^23^, ^79^ The choice of TIP4P as the water model was a considered decision. Previous work on rIAPP has shown this force field/water model combination to accurately predict its chemical shift values, resulting in TIP4P superseding TIPs3P^80^ (a CHARMM‐specific version of TIP3P), which the force field was parameterized with.^11^

The GROMOS54a7^45^ force field developed with the SPC water model^46^ was a fairly accurate force field/water model combination for predicting the Cα secondary chemical shifts of rIAPP, slightly overpredicting the α‐helical propensity of the Ala5–Ser19 region. This force field is a recent reparameterization, developed to correct the underestimation of the α‐helical propensity seen in previous versions of the GROMOS force field. Our findings show that this reparameterization significantly improves the predictions for rIAPP reported by Hoffmann et al.^11^ with the GROMOS 53a6 force field.

The AMBERff03w was chosen as it was previously tested with both rIAPP and hIAPP.^11^, ^55^ This force field is a redevelopment of Amberff03, with slight modifications to the backbone dihedral potentials to better work with the high‐quality TIP4P/2005 water model, which was reported to be effective for folded and unfolded proteins as well as IDPs.^36^ Despite previous reports, this force field does not accurately predict the Cα secondary chemical shifts of rIAPP, particularly for the central region. This effect was also seen by Hoffmann et al.,^11^ with underestimation of the α‐helical propensity of the central region.

AMBERff99SB*‐ILDN^37^, ^38^, ^39^, ^40^ was not developed for IDPs but has previously been reported to accurately represent IDPs.^11^, ^23^, ^81^, ^82^ This force field is a reparameterized version of the Amberff99sb force field, aimed at improving the torsional potentials of the backbone and side chains and correcting the previously observed bias in α‐helical representation. The Cα secondary chemical shift data reported in Figures 2 and 3 indicate that this force field had limited agreement with experimental chemical shifts for rIAPP, particularly in the central region.

A recent focus on IDPs has expanded the development of force fields to address the over‐stabilization of structure in IDPs. Recent research has shown the benefits of a grid‐based energy correction map (CMAP) that revises the main chain dihedral parameters of the disorder promoting residues G, A, S, P, R, Q, E, and K. This correction has aided the reparameterization of multiple AMBER, CHARMM, and OPLS force fields to better predict the properties of IDPs.^69^, ^83^, ^84^, ^85^, ^86^, ^87^, ^88^, ^89^ Limited testing has been done using IAPP with these corrections, with some work indicating overestimation of helical content (CHARMM22‐CMAP), whereas others see an accurate representation of IAPP NMR observables (AMBER‐ESFF1). Future studies should focus on the comparison of AMBERff99SBnmr2 with other newly developed force fields parameterized to better represent IDPs.

We also aimed to provide insight into the structural features of rIAPP and hIAPP to understand better the structural features that affect these peptides' aggregation and disease potential. The α‐helical propensity of hIAPP and rIAPP at the N‐terminal region of the peptides is consistent with multiple other experimental and computational studies.^14^, ^15^, ^22^, ^70^ The sequences of hIAPP and rIAPP are identical up to residue 18, which is an Arg in rIAPP and a His in hIAPP. The predicted Cα secondary chemical shifts and percentage secondary structure are indeed very similar until residue 18 (Figures 5 and 6), The hIAPP sequence then exhibits higher α‐helical structural propensity towards the C‐terminal region, whilst the C‐terminal region in rIAPP remains unstructured, which is consistent with the previous reports.^18^, ^22^, ^70^ It is important to point out that Reddy et al. noted that along with the Thr9–Ser19 α‐helical structure, hIAPP has a propensity to form β‐sheets at the C‐terminal end in Gly24–Ser28 and Asn31–Asn35. The secondary structure analysis (Figure 6) reveals a higher β‐sheet propensity in hIAPP than rIAPP, but helical conformations are predicted to be more likely for this region. At the same time, the free energy landscape (Figure 7) shows a more favorable (lower) free energy for higher values of β‐RMSD in hIAPP than in rIAPP.

Two distinct hydrophobic regions are present in hIAPP Leu12–Leu16 (LANFL), and Phe23–Leu27 (FGAIL), and both are thought to influence the aggregation propensity of the peptide. The chemical shift and secondary structure predictions (Figures 5 and 6) show that hIAPP and rIAPP differ mainly in the second hydrophobic region (Leu23–Pro29). The FGAIL region is the smallest fragment of hIAPP that has been shown to aggregate (GAIL did not show any aggregation).^90^ This region in rIAPP contains two proline residues, is directly preceded by a proline, and shows considerable variation of upfield and downfield Cα secondary chemical shifts. Secondary structure predictions indicate that this region is primarily random coil. This pronounced difference in secondary structure between hIAPP and rIAPP is congruent with previous reports that the proline substitutions in rIAPP end the transient α‐helix at Leu23, create a more disordered C‐terminus than that seen in hIAPP.^18^, ^19^ These differences in α‐helical propensity between hIAPP and rIAPP have been proposed to be the reason for the substantially larger aggregation tendency of hIAPP compared with rIAPP.^6^, ^19^, ^20^ In pramlintide, these three proline substitutions reduce aggregation propensity and destabilize the formation of long β‐sheet fibrils but do not entirely abolish fibril formation.^72^, ^91^ Phe23 is necessary for the aggregation of hIAPP, with studies showing that the FGAIL fragment exhibits no aggregation when Phe23 is mutated.^73^, ^90^ Our simulations predict hIAPP to have 23% helical and 23% β‐sheet propensities for this residue, which is not predicted for rIAPP, with only 2% helical and 7% β‐sheet propensities (Figure 4), which could be influential for aggregation.

The secondary structure analysis (Figure 6) predicts Val26 in rIAPP to have 93% random coil content, but this is substantially reduced in Ile26 in hIAPP to 47%. This is concomitant with increases in both helical (28%) and β‐sheet content (19%). It has been reported that mutation of Val26 in rIAPP to isoleucine results in the fastest formation of fibrils compared with the single point mutations Arg18His and Leu23Phe.^73^ Substitution of Ile26 by a proline has also been shown to reduce aggregation of wild‐type hIAPP when combined in a 1:1 ratio.^92^

The chemical shifts and secondary structure analysis (Figures 5 and 6) also show that His18 in hIAPP has a significant increase in its predicted α‐helical propensity (22%) compared with that in rIAPP (8%). It has previously been thought that this His does not play a vital role in aggregation, supported by the fact that residues 1–20 in rIAPP and hIAPP fragments form similar fibrils.^93^, ^94^ However, the mutation Arg18His has been shown to increase the aggregation propensity of rIAPP.^73^ Histidines have also been important in amyloid β fibrillogenesis.^95^, ^96^, ^97^ An increasing number of reports have implicated α‐helical structures in the early stages of amyloid aggregation. Current understanding of IAPP supports the view that the peptide is mainly in a random coil conformation, but α‐helical propensity dominates over β‐sheet propensity in monomeric amylin, which is consistent with our findings (Table 3).

One hypothesized mechanism of aggregation suggests that it occurs through the interaction of an α‐helical region in one monomer with an α‐helical region of another monomer embedded in a phospholipid bilayer.^98^ Another hypothesis suggests that the maturation into fibrils progresses through α‐helical structures before the N‐terminal α‐helical conformations are lost to form the β‐sheet structures seen in fibrillar hIAPP.^99^ Our results show that the α‐helical propensity of hIAPP extends across the entire length of the peptide, whereas in rIAPP it ends around Asn21, supporting the proposition that α‐helical conformations could either interact with a phospholipid bilayer or be the precursor to the β‐sheet aggregation process. Recent electron microscopy studies have shown that within the stacked β‐sheet structure of hIAPP fibrils, the first hydrophobic region (LANFL) is involved in forming β‐sheets between peptides. Our chemical shift and secondary structure data show a slight decrease in α‐helical propensity in hIAPP in the LANFL region that is not evident in rIAPP. This decrease could indicate the different structural features of the region relating to the other aggregation tendencies of these peptides. The second hydrophobic region (FGAIL) has been shown to cause the stacking of β‐sheets that allow the progression into fibrils,^100^, ^101^ suggesting that the mutations in rIAPP within the second hydrophobic region (FGAIL) could prevent the progression to higher order oligomers and fibrils. The second hydrophobic region in monomeric hIAPP may have two roles that differ from rIAPP, initially being involved in the α‐helical precursor to aggregation before being involved in the stacking of β‐sheets in subsequent stages of aggregation.

## 11 CONCLUSIONS

Characterization of the structure of IAPP in solution is of uttermost importance in understanding the molecular mechanisms that lead to the formation of IAPP fibrils associated with T2D. Understanding the rat form of IAPP will provide useful insights into the reasons why hIAPP can aggregate, whilst rIAPP does so to a much lesser extent. This study focused on understanding the influence of the choice of force field and water models when using MD simulation approaches to characterize the conformational behavior of the monomeric forms of rIAPP and hIAPP in aqueous solution.

NMR Cα secondary chemical shifts were predicted, and comparison with experimental values for rIAPP was used to assess the accuracy of each force field and water model combination. The region Ala5–Ser19 is observed experimentally to have a low α‐helical propensity, and hence it is of particular interest to predict this behavior accurately by confirming its greater α‐helical propensity compared with the rest of the peptide. OPLS‐AA/L with TIP4P and AMBERff99SBnmr2 with TIP4Pd were the best performing force fields at accurately predicting the Cα secondary chemical shifts of the entire peptide structure. AMBERff99SB*nmr2 with TIP4Pd was the best at predicting the α‐helical propensity of the Ala5‐Ser19 region and, therefore, appears to be the best force field for the molecular simulation of IAPP, which was indeed performed for hIAPP. Future studies should expand the range of force fields tested, and compare AMBERff99SB*nmr2 to other newly developed IDP‐specific force fields.

A comparison of the predicted Cα secondary chemical shifts of rIAPP and hIAPP confirmed that rIAPP is more disordered than hIAPP due to several proline residues. Computation of conformational free energy landscapes revealed that hIAPP has a flatter energy landscape with a higher preference for α‐helical conformations than rIAPP. Secondary structure analysis confirms that both peptides exhibit predominantly random coil conformation (hIAPP 52.4% and rIAPP 67.2%), but also that hIAPP has a more significant percentage of α‐helical content (20.0%) compared with rIAPP (11.0%). rIAPP is seen to exhibit minimal α‐helical content after residue Asn22, whereas in hIAPP α‐helical content extends across the length of the peptide. This is consistent with protein aggregation studies that have previously suggested that the aggregation mechanism may involve an initial α‐helical structure in hIAPP.

## AUTHOR CONTRIBUTIONS

Sandra J. Moore conducted and analyzed all simulations and wrote the article. Ricardo L. Mancera designed the research and proofread the article. Evelyne Deplazes proofread the article. All authors have given approval to the final version of the article.

### PEER REVIEW

The peer review history for this article is available at https://publons.com/publon/10.1002/prot.26432.

## Supporting information


**Appendix S1** Supporting Information

## Data Availability

The data that support the findings of this study are openly available in Zenodo at https://doi.org/10.5281/zenodo.6672863.
