# Supplementary material for: Influence of force field choice on the conformational landscape of rat and human islet amyloid polypeptide
Source: Proteins. 2022 Oct 7;91(3):338–53. doi: 10.1002/prot.26432 (PMC10092333; doi:10.1002/prot.26432)
Supplement: Supplementary file 1 — Appendix S1 Supporting Information [file PROT-91-338-s001.docx]

**SUPPORTING INFORMATION**

Influence of Force Field Choice on the Conformational Landscape of Rat and Human Islet Amyloid Polypeptide

Sandra J. Moore^1^, Evelyne Deplazes^1§^ and Ricardo L. Mancera^1^*

1 Curtin Medical School, Curtin Health Innovation Research Institute, Curtin Institute for Computation, Curtin University, GPO Box U1987, Perth WA 6845, Australia

§ School of Molecular Biosciences, The University of Queensland, St Lucia, QLD, 4072, Australia

Keywords: islet amyloid polypeptide (IAPP), intrinsically disordered proteins, metadynamics, secondary structure

# Convergence of free energy calculations

Convergence was assessed by comparison of the of the predicted conformational free energy landscapes over time. Figure S1 is an example of the approaches used to assess convergence using the Amberff03w force field with TIP4P/2005 water potential with rIAPP.

To confirm convergence of the free energy landscape, a free energy profile was produced approximately every 100 ns for each replica. The total volume under the curve was calculated and the difference between each time progression is plotted in Figure S1. A plot was produced for each replica to confirm each had converged and two produced for the replica biasing both CVs to demonstrate that each CV had converged. Convergence was deemed to have been reached once there were minimal fluctuations between each time progression. As an example, for this system convergence was reached after 500 ns in each replica, and the time period 500-1200 ns was then used for the secondary structure analysis.


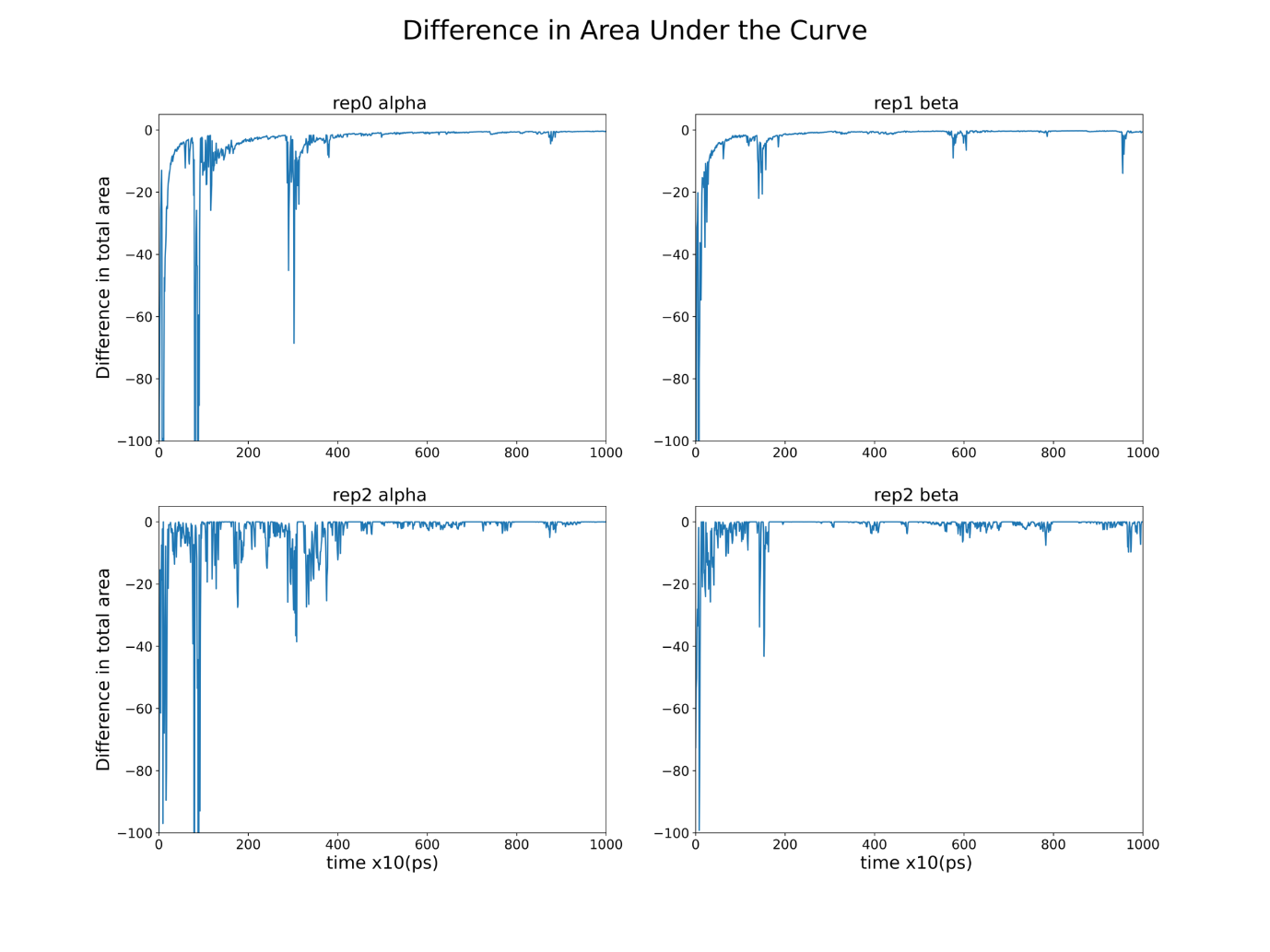


Figure S1. Assessment of convergence of the predicted free energy. The difference in the total area between each 100 ns time block of simulation are shown for each replica. Here rep0 is biased towards α-RMSD, rep1 is biased towards Β-RMSD and rep2 is biased towards both α-RMSD and β-RMSD.

# Free energy landscapes

Figure S2 shows the free energy landscapes for all the force fields tested in this work. All landscapes show the darkest purple region in the lower range of CVs, indicating the most favourable conformations are random coil.


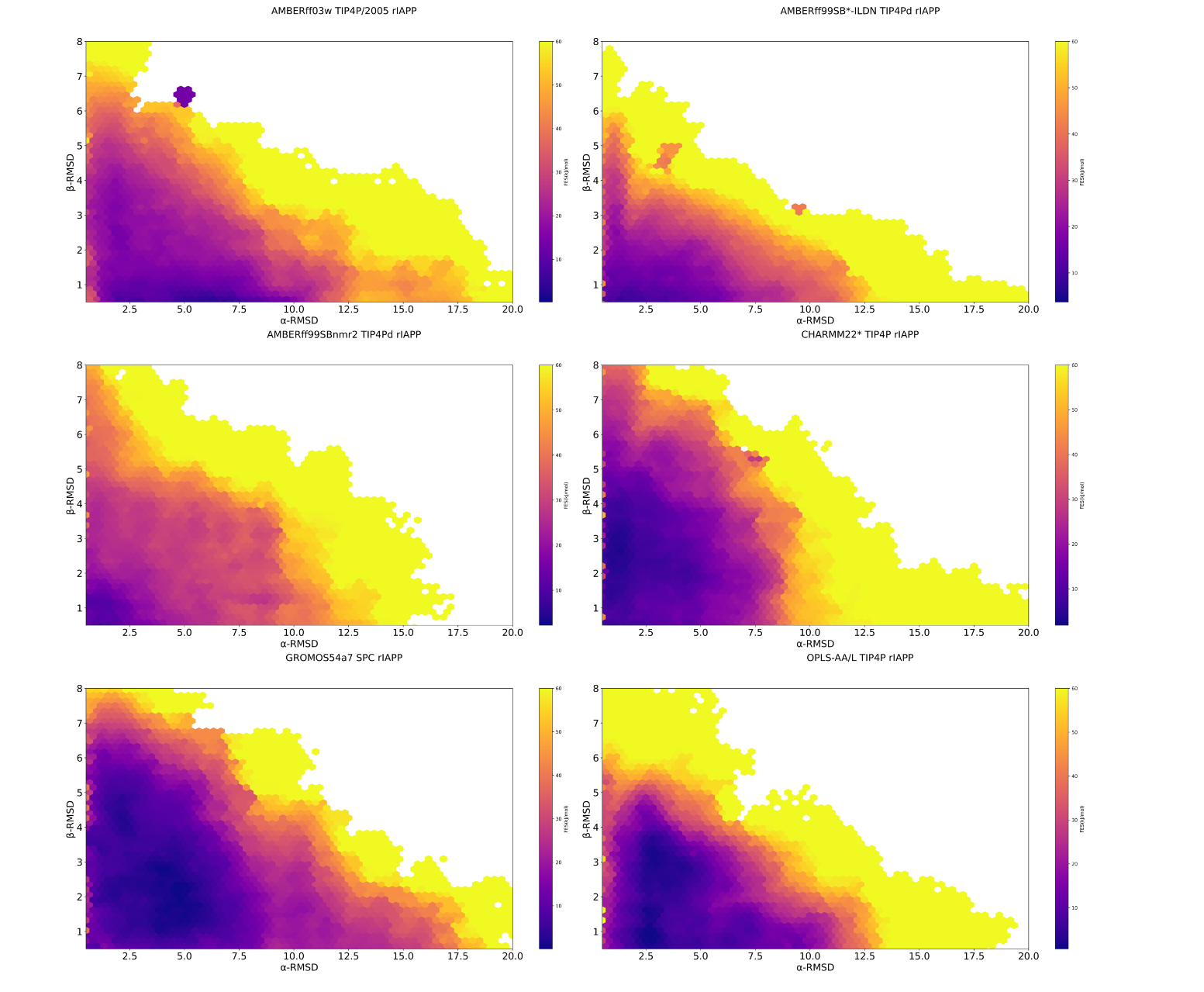


Figure S2. Conformational free energy landscapes of rIAPP for the five least accurate force fields. Free energies are shown as a function of two collective variables: α-RMSD on the x-axis and β-RMSD on the y-axis. The darker regions indicate lower free energy.

# NMR chemical shifts

**Table S1** reports the Pearson’s correlation coefficients for all NMR chemical shifts calculated for each force field. The C, Cα and Hα show the best agreement to experiment across all force fields with the highest persons correlation coefficients. The Cβ NMR chemical shifts show little agreement with experimental values, as there is little to no β-sheet content in rIAPP, and consequently the values are very small, increasing the margin for error. AMBERff03w with TIP4P/2005 and AMBERff99sb*-ILDN withTIP4Pd consistently result in the lowest correlation to experiment across all chemical shifts. AMBERff99SBnmr2 with TIP4Pd and OPLS-AA/L with TIP4P show the highest correlation across all chemical shifts, with AMBERff99SBnmr2 performing better in the region with known helical propensity (5-19), which influenced the decision to further utilise this force field to characterise hIAPP.

|  | Cα 5-19 | Cα 1-37 | Cβ | C | H | Hα |
| --- | --- | --- | --- | --- | --- | --- |
| AMBERff03w TIP4P/2005 | 0.183 | 0.634 | 0.090 | 0.732 | 0.711 | 0.689 |
| AMBERff99sb*-ILDN TIP4Pd | 0.005 | 0.620 | 0.281 | 0.761 | 0.532 | 0.809 |
| AMBERff99SBnmr2 TIP4Pd | 0.827 | 0.809 | 0.152 | 0.906 | 0.693 | 0.771 |
| CHARMM22* TIP4P | 0.751 | 0.775 | 0.432 | 0.804 | 0.769 | 0.806 |
| GROMOS54a7 SPC | 0.468 | 0.782 | 0.398 | 0.863 | 0.623 | 0.792 |
| OPLS-AA/L TIP4P | 0.634 | 0.855 | 0.523 | 0.907 | 0.661 | 0.838 |

Table S1. A comparison of each force field’s Pearson’s correlation coefficient for all chemical shifts.

# DSSP percentage secondary structure

**Table S2** reports the percentage secondary structure of each element predicted for each force field for rIAPP. All force fields predict random coil to be the most dominant conformation, which is expected due to the intrinsically disordered nature of rIAPP. All force fields predict similar trends, with turn and helix having similar percentages, and β-sheet being the least populated secondary structure.

Table 2. Percentage secondary structure (DSSP) content in rIAPP.

|  | Coil | Turn | Helix | Sheet |
| --- | --- | --- | --- | --- |
| AMBERff03w TIP4P/2005 | 59.88 | 16.74 | 13.55 | 9.72 |
| AMBERff99sb*-ILDN TIP4Pd | 69.50 | 13.03 | 15.06 | 2.28 |
| AMBERff99SBnmr2 TIP4Pd | 67.18 | 13.75 | 11.04 | 8.01 |
| CHARMM22* TIP4P | 63.67 | 12.58 | 11.24 | 9.78 |
| GROMOS54a7 SPC | 58.18 | 15.20 | 16.63 | 9.92 |
| OPLS-AA/L TIP4P | 66.88 | 14.56 | 12.94 | 5.61 |
